# Cervical Laminoplasty for Multilevel Cervical Myelopathy

**DOI:** 10.4061/2011/241729

**Published:** 2011-10-02

**Authors:** Murali Krishna Sayana, Hassan Jamil, Ashley Poynton

**Affiliations:** Department of Orthopaedics, Cappagh National Orthopaedic Hospital, Finglas, Dublin 11, Ireland

## Abstract

Cervical spondylotic myelopathy can result from degenerative cervical spondylosis, herniated disk material, osteophytes, redundant ligamentum flavum, or ossification of the posterior longitudinal ligament. Surgical intervention for multi-level myelopathy aims to decompress the spinal cord and maintain stability of the cervical spine. Laminoplasty was major surgical advancement as laminectomy resulted in kyphosis and unsatisfactory outcomes. Hirabayashi popularised the expansive open door laminoplasty which was later modified several surgeons. Laminoplasty has changed the way surgeons approach multilevel cervical spondylotic myelopathy.

## 1. Introduction

Chronic compression of the cervical spinal cord leads to a clinical syndrome of cervical spondylotic myelopathy (CSM). In degenerative cervical spondylosis, herniated disk material, osteophytes, redundant ligamentum flavum, or ossification of the PLL (OPLL) can all cause spinal cord compression. The effect on spinal cord compression is much more pronounced if a patient has a congentially small spinal canal. The aetiopathogenesis, clinical manifestations, investigations, and nonoperative management are discussed in other articles of this special edition. 

The primary aims of surgical intervention for multilevel myelopathy are to decompress the spinal cord and maintain stability of the cervical spine. Secondary aims are to minimize complications which include long-term pain and motion loss. This can be achieved by anterior approach and/or posterior approach to cervical spine. Anterior approach would involve multilevel discectomy at times with corpectomy and fusion. Posterior approach would involve laminectomy with or without fusion or laminoplasty. This article will focus on cervical laminoplasty for multilevel myelopathy. 

## 2. Evolution of Laminoplasty

Kirita, in 1968, devised a sophisticated operative technique, in which the laminae were thinned and divided at the midline using a high-speed drill followed by their en bloc resection to achieve total decompression of the compressed spinal cord [1]. Although this technique improved the results and bettered the technique of conventional laminectomy, complications like postoperative kyphosis and membrane formation resulted. Oyama et al. reported a Z-plasty (Hattori technique) of the cervical spine laminae in 1973 [2]. This procedure was technically demanding and had not been adopted widely other surgeons. In the year 1977, Hirabayashi et al. described an expansive open door laminoplasty (ELAP), which is a relatively easier and safer procedure than laminectomy [3]. He utilized and modified Kirita's technique of using a high-speed drill and started doing en bloc laminectomies by drilling bilateral bony gutters at the junction of laminae and facet joints. He conceptualized the idea of ELAP, when he noticed the presence of dural pulsation, a sign of decompression of the dura, after he lifted just one side of the laminae just before their complete removal. He used this technique to manage OPLL, which is relatively frequent amongst Japanese. This technique is now indicated in surgical management of multilevel cervical spondylotic myelopathy. It has eliminated the postoperative complications of laminectomy by preserving the posterior elements, thereby avoiding the postoperative malalignment of the cervical spine, postlaminectomy membrane, late neurologic deterioration and instability, and vulnerability of the spinal cord caused by total removal of the posterior structures. The laminae are still available for stability and for the attachment of the paraspinous muscles. In biomechanical studies with human and animal models, a spine treated with laminoplasty is comparable to the intact spine [4, 5]. 

## 3. Indications

Indications for laminoplasty include multilevel cervical stenosis and myelopathy, preferably with stenosis at 3 or more levels [7]. If there is segmental instability, this can be addressed with a concurrent lateral mass fusion of the involved levels. Major contraindications include the presence of kyphosis and preoperative neck pain. Neck pain can be a significant complication of laminoplasty, and this can be minimized by treating patients with neck pain with a concomitant posterior lateral mass fusion or by performing an anterior decompression and fusion instead. In order to ensure the best results, patients with significant neck pain should not be treated with a laminoplasty. 

## 4. Surgical Technique

**Figure 1:**
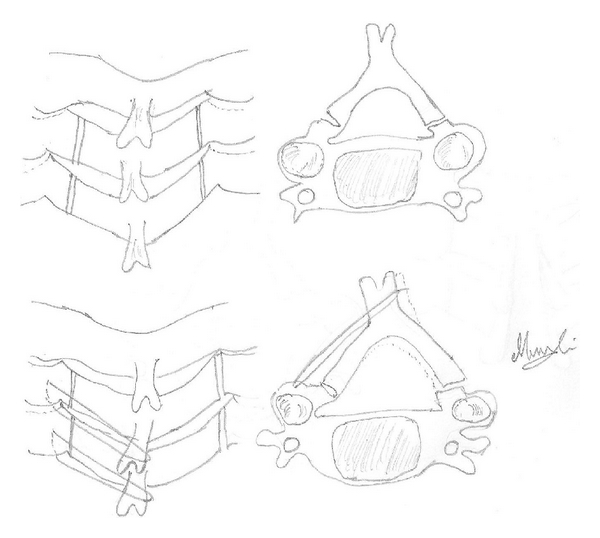
(a) The laminae which are bilateral troughs (complete on one side and incomplete on the other) made at the junction of laminae and lateral masses. (b) Greensticking on the incomplete trough side so that spinal canal is now expanded. Figures redrawn from Steinmetz and Resnick [6].

ELAP is a canal-expanding procedure in which the laminae are elevated on one side and bent or “greensticked” on the other (Figure 1). After obtaining an informed consent, under general anaesthetic, the patient is positioned prone, on a surgical table, which is tilted approximately 30 degrees cranially upwards. The head securely fixed with a Mayfield fixator which is firmly attached to the surgical table. After prepping and draping the neck, through a midline incision, tips of the spinous processes are exposed. Bilateral paracervical muscles are stripped off from the laminae usually between C3 and C7 by cautery or a periosteal elevator. The open side gutter is burred at the junctions of the laminae and facet joints by a high-speed cutting burr. The ventral cortex is either excised with a thin-bladed Kerrison rongeur or perforated with a diamond burr. The ligamentum flavum at the upper and lower ends of the laminar door, usually at C2/3 and C7/T1 are resected with a thin-bladed Kerrison rongeur. Then, the bony gutter on the hinge side is prepared with slightly more lateral than the other side. The laminar door is ready to be opened, when all spinous processes and laminae become slightly mobile yet retain a spring-like resistance. Pieces of suture are placed through the facet joint capsule and surrounding soft tissues at each level in the hinge side and are passed through interspinous ligaments around the base of the corresponding spinous process. Just prior to opening the laminar door, the patient's neck position is converted from a flexed to a neutral position. The tip of the blade of a large Kerrison rongeur is placed under the excised margin of one lamina and its edge is lifted slightly. The spinous process is held in the expanded position by fingers of an assistant. Then the next lamina is lifted in the same manner until all laminae are opened to the same extent. Repeat this procedure slowly and open the laminar door gradually. Release adhesions between the laminae and the dura with a spatula as needed. The hinge may break, if a lamina extensively opened at any given time. Usually, dural pulsation can be observed in the middle of the opening procedure even before complete expansion. These maneuvers should be continued until the laminae of the open side become almost horizontal. To maintain the decompressed position and to prevent the reclosure, threads previously placed at the base of the spinous processes are securely tied. The bilateral neck muscles are approximated to minimize the dead space, and the nuchal ligament is tightly closed with nonabsorbable sutures [8]. Postoperatively, the patient is ambulated at the third postoperative day with a soft collar, which is worn for an average of a week. After its removal, the patient is encouraged to start gentle ROM exercise of the neck. Stitches are removed at 10 days postoperatively. The patients usually return to their work after 3 to 4 weeks. Rigorous activities including sports are permitted after 3 months postoperatively.

## 5. Modifications of ELAP

**Figure 2:**
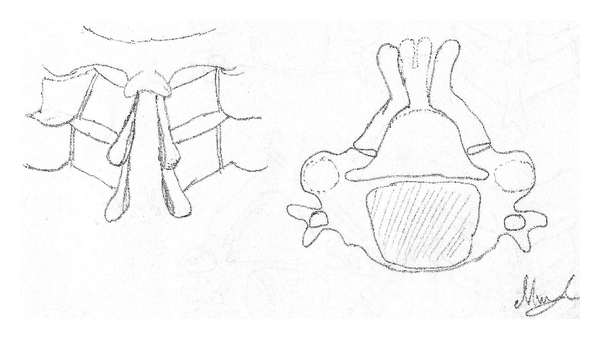
French door style of laminoplasty, the spinous process is split in the midline and the laminae hinged bilaterally, thereby expanding the spinal canal. Figures redrawn from Steinmetz and Resnick [6].

Itoh and Tsuji introduced the concept of the en bloc laminoplasty in which the laminae are propped open with bone graft at every other level to prevent closure of the laminoplasty [9]. Other techniques have been described including spinous process splitting procedures (Figure 2; French door laminoplasty—FDL, Kurokawa modification of FDL) [10]. It is important to reattach the nuchal muscles to the C2 spinous process in order to maximize postoperative function.

## 6. Results

Outcomes for cervical myelopathy are measured by Japanese Orthopaedic Association scoring (Table 1) system which scores upper limb, lower limb function, sensory system, and sphincter function [11]. Maximum score possible (normal function) is 17. This scoring system is widely used to report the improvement following surgical intervention for CSM. The overall recovery rate reported varies from 50% to 70% [6]. The degree of preoperative myelopathy seems to determine the extent of postoperative recovery and the extent of recovery is not related to the specific surgical procedure. There is no evidence that one surgical technique has been proven to be more effective than another. Miyazaki et al. observed that improved neurologic status was maintained at a mean of 12 years after surgery [12]. 

Worsening of postoperative spinal alignment has been reported to vary from 22% to 53%, this does not include kyphosis [6]. The incidence of postoperative kyphosis varies from 2%–4% [6]. There is no standard of measuring and reporting the preoperative and postoperative cervical alignment and it is, therefore, difficult to compare one paper with other. Cervical ROM has been reported to decrease 17–50%, with an average of approximately 50% after laminoplasty. When laminoplasty is augmented with fusion, ROM was decreased 70–80%. 

Liu et al. in 2009 reported a 9.2% revision surgery rate at 10-year followup of 130 patients who underwent cervical laminoplasty [13]. They classified laminoplasty failures into 3 categories: “technique related,” “inadequate symptomatic relief after treatment,” or “recurrence of symptoms due to disease progression. Disease progression accounted for the largest group of revised patients (66%) who had a surgical revision risk of 4% ± 2.2% and 21% ± 7.7% at 1 and 4 years following the original laminoplasty. These numbers are comparable to those following fusion operations. One avoidable cause of revision surgery was the failure of suture anchors, as newer techniques such as plating of laminoplasties avoided revisions for laminar reclosure. The typical surgeries performed as revision procedures are laminectomy and posterior/anterior fusion, anterior cervical decompression and fusion (ACDF), or circumferential fusion.

## 7. Complications

One complication specific to laminoplasty is axial symptoms which include shoulder pain, shoulder spasm, and neck pain. The postoperative incidence could be as high as 60% [14]. Therefore, it is important that case selection for laminoplasty should specifically identify preoperative neck pain and avoid laminoplasty in these cases. 

Another complication that may be found with laminoplasty is C5 nerve root paresis manifesting as deltoid paralysis and biceps weakness with a reported incidence varying from 3%–11% [3, 15]. This tends to improve over a 6-month period. The reason for this complication is the traction on C5 nerve roots as there is an acute posterior shift of the spinal cord following decompression and C5 nerve roots are most commonly affected. Laminectomy with lateral mass fusion is indicated in patients with preoperative axial symptoms and instability. 

## 8. Minimal Invasive Methods

Minimal invasive technique has been reported by Benglis et al., who have explored the feasibility of using minimal invasive techniques to perform the open door laminoplasty [16]. They used tubular dilator retractors and gained experience on six cadavers. They have reported a successful intervention in a patient with acute central cord syndrome with critical cervical stenosis. They claim that this was technically challenging and took twice the time required for an open procedure. They believe that by leaving the midline muscular and tendinous attachments intact, incidence of postoperative axial symptoms would decrease.

## Figures and Tables

**Table 1:** Evaluation system for cervical myelopathy (compiled by the Japanese Orthopedic Association [11]).

Section score	
	Points

(I) Upperextremity function	
Impossible to eat with either chopsticks or spoon	0
Possible to eat with spoon, but not with chopsticks	1
Possible to eat with chopsticks, but inadequate	2
Possible to eat with chopsticks, but awkward	3
Normal	4
(II) Lower extremity function	
Impossible to walk	0
Need cane or aid on flat ground	1
Need cane or aid on stairs	2
Possible to walk without cane or aids, but slow	3
Normal	4
(III) Sensory	
A Upper extremity	
Apparent sensory loss	0
Minimal sensory loss	1
Normal	2
B Lower extremity	
Apparent sensory loss	0
Minimal sensory loss	1
Normal	2
C Trunk	
Apparent sensory loss	0
Minimal sensory loss	1
Normal	2
(IV) Bladder function	
Urinary retention or incontinence	0
Severe dysuria (sense of retention, straining)	1
Slight dysuria (pollakiuria, retardation)	2
Normal	3

Normal condition = total of best score (I + II + III + IV) = 17 points.
